# Probable posttransplant lymphoproliferative disorder after pediatric living donor liver transplantation: Is a biopsy still needed?

**DOI:** 10.1002/ccr3.6454

**Published:** 2022-11-04

**Authors:** Muneyuki Matsumura, Shigehito Miyagi, Kazuaki Tokodai, Toshiaki Kashiwadate, Atsushi Fujio, Koji Miyazawa, Kengo Sasaki, Yoshikatsu Saito, Norifumi Kanai, Michiaki Unno, Takashi Kamei

**Affiliations:** ^1^ Department of Surgery Tohoku University Graduate School of Medicine Sendai Japan

**Keywords:** pediatric living donor liver transplantation, preemptive therapy, probable PTLD

## Abstract

Posttransplant lymphoproliferative disorder (PTLD) is a complication of solid organ transplantation and is associated with Epstein‐Barr virus (EBV). Recently, EBV‐related PTLD was defined as probable PTLD or proven PTLD. Probable PTLD involves significant lymphadenopathy, hepatosplenomegaly, or other end‐organ manifestations, without a histological diagnosis, together with significant EBV DNAemia. Proven PTLD is the detection of EBV‐encoded proteins in a tissue specimen, together with symptoms and/or signs originating from the affected organ. Probable PTLD after pediatric liver transplantation has not been well documented. Therefore, here, we aimed to describe cases of five pediatric patients with probable PTLD after liver transplantation, who were successfully treated with preemptive immunosuppression reduction with or without rituximab. All five patients (age range, 1–4 years; two girls and three boys) had EBV DNAemia. Three patients developed probable PTLD within 12 months of transplantation. Further, three patients had a significantly high EBV viral load, but the other two patients with lymphadenopathy and end‐organ manifestation had a relatively low EBV viral load. Early onset pediatric PTLD with significant EBV DNAemia is almost universally EBV‐related. Biopsy was not performed in any patient due to the relative inaccessibility of the lesion and young age of the patients. If the patient’s symptoms are too mild, if excisional biopsy is too difficult to perform, or if the patient is too sick to undergo an invasive procedure, initiating preemptive treatment without a histological diagnosis could be the treatment option.

## Graphical abstract

We had five cases of pediatric probable posttransplant lymphoproliferative disorder after liver transplantation. If the patient's symptoms are too mild, excisional biopsy is too difficult to perform, or the patient is too sick to undergo invasive procedures, initiating preemptive treatment without a histological diagnosis could be the treatment option.

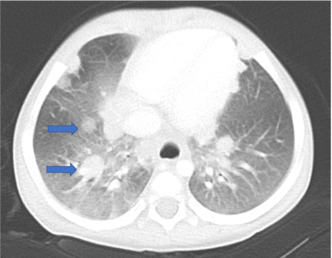

## 1 BACKGROUND

Liver transplantation is the only viable therapeutic option for children with end‐stage liver disease. There has been significant improvement in terms of the outcomes for patients and graft survival in recent decades. However, problems exist beyond short‐term complications such as hepatic artery thrombosis, portal vein thrombosis, or infection. Long‐term complications such as metabolic disorders due to immunosuppressive medications, chronic rejection, and de novo cancers need to be solved.^1^, ^2^

Posttransplant lymphoproliferative disorder (PTLD) is one of the most common malignancies occurring after solid organ transplantation (SOT) and hematopoietic stem cell transplantation (HSCT).^3^ Since it was first described in 1969 by Penn et al., PTLD was believed to be a relatively rare disorder. However, during the past 10 to 15 years, the incidence of PTLD has increased, which has been associated with high morbidity and mortality after SOT and HSCT.^4^, ^5^ PTLD encompasses a heterogeneous group of diseases that inherently have a wide range of clinicopathological characteristics, from incidental asymptomatic findings to fulminant lymphoma presenting with multiorgan failure.^6^

The characteristics of PTLD differ between pediatric and adult recipients. The following were the findings in pediatric recipients: (1) more than 70% of patients had early‐onset PTLD that occurred within the first year of transplantation^7^, ^8^ and (2) the histopathology of more than 90% of patients indicated positivity for Epstein–Barr virus (EBV) deoxyribose nucleic acid (DNA)emia and EBV‐encoded proteins.^9^ In contrary to pediatric recipients, adult recipients tend to have late‐onset PTLD and negative EBV DNAemia.^10^ EBV‐related PTLD has recently been defined as probable PTLD or proven PTLD.^11^ Probable PTLD is defined as significant lymphadenopathy, hepatosplenomegaly, or other end‐organ manifestations, without a histological diagnosis, accompanied by significant EBV DNAemia. Proven PTLD is defined as the detection of EBV‐encoded proteins in a tissue specimen, together with symptoms and/or signs originating from the affected organ.^12^

Based on the characteristics of early‐onset PTLD after pediatric liver transplantation, which is almost universally EBV‐related, we assumed that the same management could be offered for both proven and probable PTLD. In this paper, we aimed to describe five cases of probable pediatric PTLD after living donor liver transplantation (LDLT) and to review the relevant literature.

## 2 CASE SERIES

This study was approved by the Ethics Committee of Tohoku University (approval number: 2018‐1‐442) and was conducted in accordance with the institutional guidelines as well as the ethical guidelines mandated by the Declaration of Helsinki (2013).

### 2.1 Case 1

The first case involved a 4‐year‐old girl with a past medical history remarkable for biliary atresia status post‐Kasai portoenterostomy. She underwent LDLT from her mother when she was 3 years old. The donor (D) and recipient (R) pretransplant EBV serostatus at the time of LDLT was D+/R+. The postoperative course was complicated with acute cellular rejection (ACR), which was successfully treated with steroid pulse therapy and deoxyspergualin. The patient received tacrolimus and methylprednisolone (MP) for immunosuppression. Fourteen months after LDLT, she presented with pneumonia, and a contrast‐enhanced computed tomography (CT) scan showed bulky retroperitoneal lymph node enlargement. The Ann Arbor stage was I, and the viral load detected through serum EBV polymerase chain reaction (PCR) quantification at diagnosis was 26,891 copies/ml. The peak serum EBV viral load was 38,099 copies/ml. We considered retroperitoneal lymph node biopsy for a more careful examination but decided not to proceed because of the relative inaccessibility of the lesion. Immunosuppression reduction was initiated, and the serum EBV viral load slowly decreased, following which the retroperitoneal lymph node shrunk. No rejection was observed more than 30 years after immunosuppression reduction.

### 2.2 Case 2

**FIGURE 1:**
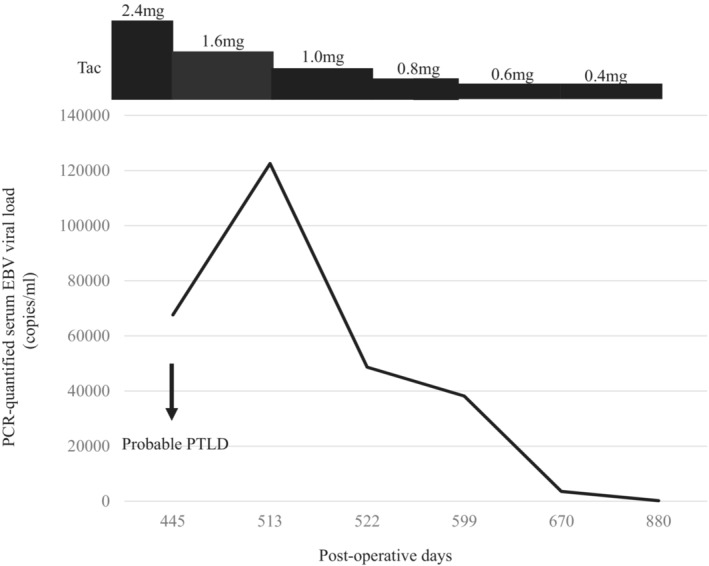
Treatment scheme of Case 2. The PCR‐quantified serum EBV viral load and dosage of Tac are shown. The dose of tacrolimus was slowly decreased to a quarter of the original dose over 6 months. The EBV viral load became undetectable in the following 6 months. EBV, Epstein–Barr virus; PCR, polymerase chain reaction; Tac, tacrolimus.

The second case involved a 2‐year‐old girl with a past medical history remarkable for biliary atresia status post‐Kasai portoenterostomy and a second anastomotic revision. She underwent LDLT from her mother when she was 11 months old. The pretransplant EBV serostatus at the time of LDLT was D+/R+. The patient's postoperative course was uneventful. The patient received tacrolimus and MP for immunosuppression. Fifteen months after LDLT, she presented with high fever, and a contrast‐enhanced CT scan revealed bulky retroperitoneal lymph node enlargement. The Ann Arbor stage was I. The patient's PCR‐quantified serum EBV viral load at diagnosis was 67,610 copies/ml. The peak serum EBV viral load was 122,539 copies/ml. Lymph node biopsy was not performed due to the mildness of the patient's symptoms, and immunosuppression reduction was started. At the diagnosis of the probable PTLD, she received only tacrolimus. We continued to administer tacrolimus but reduced the dose in half over the next 4 months. The serum EBV viral load slowly decreased but persisted over the next 15 months. The retroperitoneal lymph node shrunk thereafter. The tacrolimus dosage and EBV viral load are described in Figure 1. We continued minimum immunosuppression, and no rejection was observed more than 25 years.

### 2.3 Case 3

**FIGURE 2:**
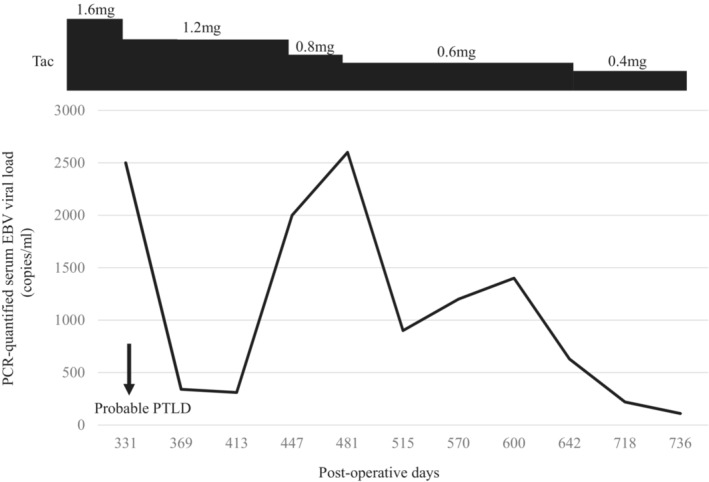
Treatment scheme of Case 3. The PCR‐quantified serum EBV viral load and dosage of tacrolimus (Tac) are shown. The dose of tacrolimus was slowly reduced by almost half over 6 months. The EBV viral load was persistent over the following year. EBV, Epstein–Barr virus; PCR, polymerase chain reaction; Tac, tacrolimus.

The third case involved a 2‐year‐old boy with a past medical history remarkable for biliary atresia status post‐Kasai portoenterostomy. He underwent LDLT from his mother when he was 9 months old. The pretransplant EBV serostatus at the time of LDLT was D+/R+. The patient's postoperative course was uneventful. The patient received tacrolimus and MP for immunosuppression. Twelve months after LDLT, he presented with fever, and a contrast‐enhanced CT scan showed multiple lung nodules. We also performed^18^F‐fluorodeoxyglucose‐positron emission tomography (FDG‐PET), which was significant for a standardized uptake value maximum (SUV max) of 1.9 in the lung nodules. The Ann Arbor stage was I. The patient's PCR‐quantified serum EBV viral load at diagnosis was 2500 copies/ml. Lung biopsy was not performed due to the difficulty in performing bronchoscopy in a 2‐year‐old boy. We started immunosuppression reduction. The tacrolimus dose was slowly reduced almost in half over the next 6 months. The EBV viral load was persistent over the next year, but the lung nodule shrunk thereafter. The tacrolimus dosage and EBV viral load are described in Figure 2. We continued minimum immunosuppression, and no rejection was observed more than 20 years.

### 2.4 Case 4

**FIGURE 3:**
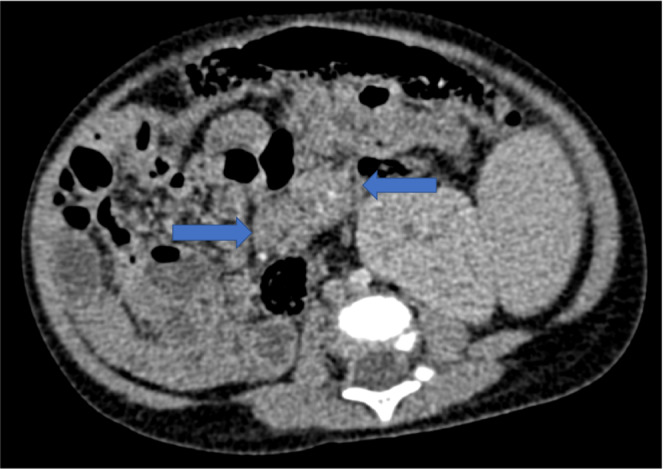
Enlarged retroperitoneal lymphadenopathies. Contrast‐enhanced computed tomography showed enlarged retroperitoneal lymphadenopathies (arrows) that were considered for excisional biopsy, but a biopsy was not performed due to the relative inaccessibility of the lesions.

The fourth case involved a 1‐year‐old boy with a history of biliary atresia status post‐Kasai portoenterostomy. He underwent LDLT from his mother when he was 11 months old. The pretransplant EBV serostatus at the time of LDLT was D+/R+. The postoperative course was unremarkable. The patient received tacrolimus and MP for immunosuppression. Three months after LDLT, he presented with diarrhea, and a contrast‐enhanced CT scan was significant for multiple retroperitoneal lymphadenopathies (Figure 3). We also performed FDG‐PET, which revealed an SUV max of 2.3 indicative of lymphadenopathy. The patient's PCR‐quantified serum EBV viral load at diagnosis was 9900 copies/ml. The Ann Arbor stage was I. We considered retroperitoneal lymph node biopsy but decided against it because of the difficulty of the procedure. Immunosuppression reduction was initiated. Tacrolimus was discontinued and MP was increased from 3 to 10 mg. For 2 weeks, the patient received no tacrolimus. After his condition improved, we resumed tacrolimus using half the dosage. No rejection was noticed throughout this admission. We continued the minimum immunosuppression, and no rejection observed for 10 years so far. The EBV viral load was decreased to 1700 copies/ml when tacrolimus was resumed. Retroperitoneal lymphadenopathy remained to a small extent, but significant uptake was not observed in a subsequent FDG‐PET scan.

### 2.5 Case 5

**FIGURE 4:**
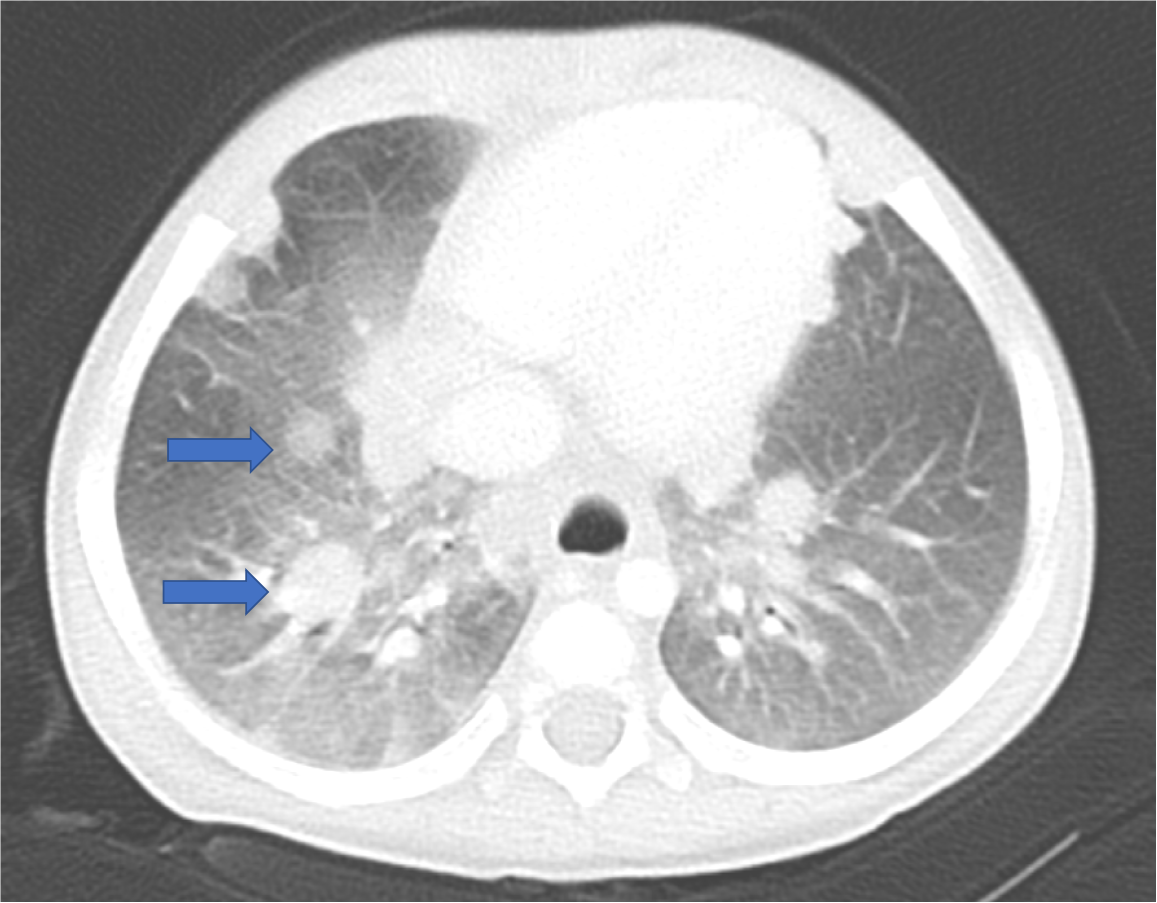
Multiple lung nodules. Contrast‐enhanced computed tomography showed multiple lung nodules (arrows) that were considered for lung biopsy, but a biopsy was not performed due to the patient's severe respiratory insufficiency.

**FIGURE 5:**
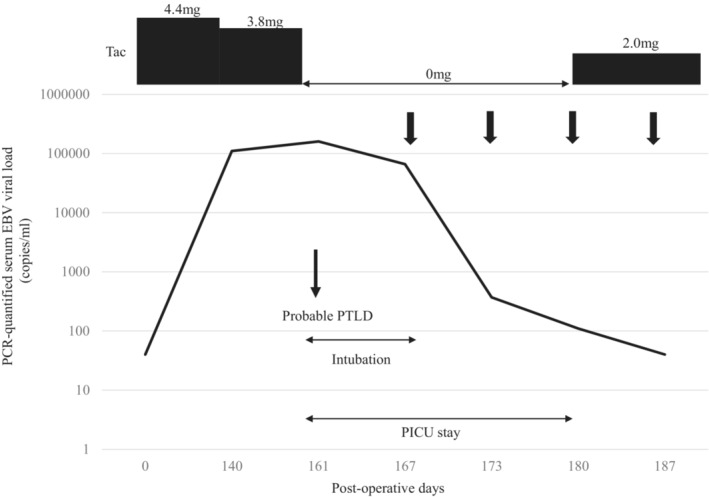
Treatment scheme of Case 5. The PCR‐quantified serum EBV viral load, tacrolimus dosage, and rituximab administration timing are shown. Changes in the PCR‐quantified serum EBV viral load in relation to the dose of tacrolimus (Tac) are presented. Thick arrows indicate the timing of rituximab administration (375 m^2^/BSA). BSA, body surface area; EBV, Epstein–Barr virus; PCR, polymerase chain reaction; PICU, pediatric intensive care unit.

The fifth case involved a 1‐year‐old boy with a past medical history remarkable for biliary atresia status post‐Kasai portoenterostomy. He received LDLT from his mother when he was 13 months old. The pretransplant EBV serostatus at the time of LDLT was D+/R+. The postoperative course was unremarkable. The patient received tacrolimus and MP for immunosuppression. Three months after his operation, MP was discontinued. Five months after LDLT, he presented with severe respiratory insufficiency and sepsis. Immediately after admission to the surgical intensive care unit, the patient was intubated. A contrast‐enhanced CT scan showed a brain mass, multiple lung nodules, mediastinal lymphadenopathy, a right kidney mass, and an intra‐abdominal mass next to the bladder (Figure 4). The patient's PCR‐quantified serum EBV viral load at diagnosis was 110,000 copies/ml. Lung biopsy was considered but was not performed due to severe respiratory insufficiency. Immunosuppression reduction was started, and the first dose of rituximab was administered once the patient's condition stabilized. In total, he received four rounds of rituximab. Serum EBV PCR quantification indicated that the viral load increased to 160,000 copies/ml at the peak level and that it decreased to undetectable levels after treatment. Due to the patient's severe condition, no tacrolimus was administered for 19 days, although we continued to administer steroids for severe respiratory insufficiency. We resumed the half dose of tacrolimus. No rejection was noticed throughout this admission. The PCR‐quantified serum EBV viral load, tacrolimus dosage, and timing of rituximab administration are described in Figure 5. We continued minimum immunosuppression for 2 years so far and no rejection was observed. A contrast‐enhanced CT scan and brain magnetic resonance imaging showed that the masses found throughout his body had shrunk.

## 3 DISCUSSION

Our case series represents a pediatric patient population with lymphadenopathy or other end‐organ manifestations with significant EBV DNAemia after liver transplantation, without a histological diagnosis (Table 1). For probable PTLD, early immunosuppression reduction and even preemptive rituximab therapy could be the treatment option. Probable PTLD was defined as significant lymphadenopathy, hepatosplenomegaly, or other end‐organ manifestations, without a histological diagnosis, together with significant EBV DNAemia.^12^, ^13^ Proven PTLD was defined as the detection of EBV‐encoded proteins in a tissue specimen, together with symptoms and/or signs originating from the affected organ. In previous studies on large patient cohorts with PTLD after SOT, probable PTLD was not well described or was not recorded as PTLD.^14^, ^15^, ^16^ However, in the case of PTLD after HSCT, Styczynski et al.^5^ concluded that classifying the diagnosis as proven or probable had no effect on PTLD‐related mortality (35.38% vs. 24.14%, *p* = 0.2). Based on this novel study, we believe that probable PTLD in pediatric liver transplantation recipients is worth reporting.

**TABLE 1:** Patient characteristics, perioperative variables, and PTLD‐related variables

No.	Underlying diseases	Age at transplantation (months)	Time (month) from transplantation to the diagnosis of PTLD	EBV serostatus (D/R)	Acute rejection before PTLD	EBV viral load at diagnosis	EBV viral load at peak	Symptom/signs of PTLD	Primary site of PTLD	Ann Arbor stage	Reason for not performing biopsy	Treatment	Clinical outcome
1	Biliary atresia	46	14	+/+	Yes	26,891	38,099	Pneumonia	Retroperitoneal lymph node	I	Inaccessibility of the lesion	Immunosuppression reduction	Recovery without EBV DNA‐emia/shurunken lymph node
2	Biliary atresia	11	15	+/+	No	67,610	1,22,539	Fever	Retroperitoneal lymph node	I	Mildness of the symptom	Immunosuppression reduction	Recovery without EBV DNA‐emia/shurunken lymph node
3	Biliary atresia	9	12	+/+	No	2500	2600	fever	lung nodules	I	Inaccessibility of the lesion	Immunosuppression reduction	Recovery without EBV DNA‐emia/shurunken lung nodules
4	Biliary atresia	11	3	+/+	No	9900	9900	Diarrhea	Retroperitoneal lymph node	I	Inaccessibility of the lesion	Immunosuppression reduction	Recovery without EBV DNA‐emia/remaining lymphadenopathy
5	Biliary atresia	11	5	+/+	No	1,10,000	1,60,000	ARDS	Brain mass/lung nodules/mediastinal lymph nodes/ right kidney mass/ intra‐abdominal mass	IV	Sickness to undergo invasive procedure	Immunosuppression reduction/ four rounds of rituximab	Recovery without EBV DNA‐emia/shurunken mass

PTLD after pediatric liver transplantation has been reported to have an incidence of 6% to 20% and a mortality of 12%–60%.^17^ A younger age and EBV naivety in recipients at the time of transplantation are known to be significant risk factors for developing PTLD. Early‐onset PTLD, which develops within 1 year after transplantation, occurs mainly in pediatric recipients. The general concept is that early‐onset PTLD has favorable outcomes, while late‐onset PTLD is thought to behave like a more aggressive lymphoma. In addition, EBV‐negativity results in a worse prognosis than early‐onset PTLD.^18^ Several case series have shown that early‐onset PTLD in pediatric recipients is almost universally EBV‐related^7^, ^19^ contrary to many PTLD‐related conditions in adult recipients, which are not EBV‐related.^20^ Therefore, pediatric recipients generally develop early PTLD, which is EBV‐related, resulting in a relatively favorable prognosis. In our case series, three patients developed probable PTLD within 12 months of LDLT, which is consistent with previously reported data.^7^, ^19^ All five patients had EBV DNAemia. In terms of disease onset and EBV serostatus, our case series represents the general population of patients with PTLD after pediatric liver transplantation, even though the diagnosis was probable PTLD.

In our case series, four patients had retroperitoneal lymphadenopathy, for which we decided not to perform biopsy. The reasons for not performing biopsy were the relative inaccessibility of the lesion. In the past, many studies reported that laparoscopic retroperitoneal lymph node biopsy was useful and less invasive.^21^, ^22^ However, our patients had a history of LDLT, and severe abdominal adhesions were suspected. Furthermore, our patients were too young for us to perform laparoscopy. We believe that the open laparotomy biopsy procedure is too invasive. Moreover, we believe radiological‐guided needle biopsies were too difficult for our patients and sometimes insufficient to make a diagnosis. In Case 5, the patient had acute respiratory distress syndrome, and we found that the patient developed markedly high EBV DNAemia and an Ann Arbor stage IV mass. We considered performing a biopsy for the lung mass but decided otherwise due to respiratory insufficiency. From our standpoint, the reasons for not performing biopsies in patients suspected of having PTLD are as follows: (1) patient symptoms are too mild to perform invasive procedures; (2) it is too difficult to obtain an excisional biopsy in lymphadenopathy, and (3) the patient is too sick to undergo invasive procedures. From this point of view, we assume that in pediatric cases, the incidence rate of probable PTLD should be higher than that in adult cases, which has not been reported so far.

The presence of EBV DNAemia is the most important risk factor associated with the development of PTLD after pediatric liver transplantation. However, it is well known that a chronic high viral load carrier status is common in liver transplant recipients.^23^ Many studies have reported that the cutoff value for predicting the development of PTLD ranges from 4100–145,250 copies/ml.^7^, ^24^, ^25^, ^26^ Routine EBV PCR monitoring makes it possible to perform preemptive management through immunosuppression reduction for preventing progression to PTLD; however, the cutoff value of the EBV viral load to start immunosuppression reduction is still under discussion. The same management strategy could be applied to some patients with probable PTLD. When patients have significant EBV DNAemia, lymphadenopathy, and end‐organ manifestations and if the symptoms are mild, preemptive immunosuppression reduction can be started. In our case series, three patients had a significantly high EBV viral load, but the other two patients with lymphadenopathy and end‐organ manifestation had a relatively low EBV viral load. However, Fox et al. reported that 23% of patients with PTLD occurring after HSCT had an EBV viral load of less than 10,000 copies/ml.^27^ The possibility of probable PTLD cannot be excluded based solely on the EBV viral load. We decided to start preemptive immunosuppression reduction, and all patients responded to this strategy.

Rituximab can be the treatment choice for preemptive therapy or EBV‐related PTLD if the patient does not respond to immunosuppression reduction or if the patient has severe symptoms. For preemptive therapy, the EBV viral load threshold for administering rituximab is still controversial. Some authors have set thresholds of 1000 copies/ml,^28^ 10,000 copies/ml,^29^ or 40,000 copies/ml.^30^ Furthermore, studies on pediatric patients are limited. Kobayashi et al. demonstrated the effectiveness of preemptive therapy for EBV DNAemia after pediatric HSCT.^31^ In several large cohort studies, EBV DNAemia was found to occur after HSCT and not after SOT, although this concept can be applied to PTLD after SOT. Based on several novel large cohort studies conducted after pediatric liver transplantation, which showed that PTLD is universally related to EBV, the administration of preemptive therapy with rituximab for probable PTLD could be justified. In case five, we administered a total of four rounds of rituximab without a histological diagnosis; the patient who had probable PTLD recovered, and the viral load was undetectable through EBV PCR quantification.

## 4 CONCLUSIONS

In conclusion, we have reported five cases of probable PTLD after pediatric liver transplantation that were treated with immunosuppression reduction alone or in combination with rituximab therapy. When pediatric recipients have a typical high EBV viral load along with lymphadenopathy and systemic manifestations and if the biopsy procedure is difficult, the procedure may not necessarily be required, and immunosuppression reduction could be started; further, rituximab could be the treatment of choice.

## AUTHOR CONTRIBUTIONS

MM participated in the research design, the performance of the research and the writing of the paper. SM participated in the performance of the research. KT participated in the performance of the research. TK participated in the performance of the research. AF participated in the performance of the research. KM participated in the performance of the research. KS participated in the performance of the research. YS participated in the performance of the research. NK participated in the performance of the research. UM participated in the performance of the research. TK participated in the performance of the research.

## FUNDING INFORMATION

The authors declare that there was no source of funding involved in the present study.

## CONFLICT OF INTEREST

There is no conflict of interest involved in the present study.

## CONSENT

Written informed consent was obtained from the patient to publish this report in accordance with the journal's patient consent policy.

## Data Availability

None.

## References

[1] Tokodai K , Miyagi S , Nakanishi C , et al. Association of post‐transplant donor‐specific HLA antibody with liver graft fibrosis during long‐term follow‐up after pediatric liver transplantation. Pediatr Transplant. 2018;22:e13169. 29542229 10.1111/petr.13169

[2] Miyagi S , Kakizaki Y , Shimizu K , et al. Arterial and biliary complications after living donor liver transplantation: a single‐center retrospective study and literature review. Surg Today. 2018;48:131‐139. 28439714 10.1007/s00595-017-1515-9

[3] Romero S , Montoro J , Guinot M , et al. Post‐transplant lymphoproliferative disorders after solid organ and hematopoietic stem cell transplantation. Leuk Lymphoma. 2019;60:142‐150. 29966464 10.1080/10428194.2018.1474462

[4] Penn I , Hammond W , Brettschneider L , Starzl TE . Malignant lymphomas in transplantation patients. Transplant Proc. 1969;1:106‐112. 4944206 PMC2845467

[5] Styczynski J , Gil L , Tridello G , et al. Response to rituximab‐based therapy and risk factor analysis in Epstein Barr Virus‐related lymphoproliferative disorder after hematopoietic stem cell transplant in children and adults: a study from the Infectious Diseases Working Party of the European Group for Blood and Marrow Transplantation. Clin Infect Dis. 2013;57:794‐802. 23771985 10.1093/cid/cit391

[6] Matsumura M , Mizuno Y , Okamoto M , et al. Long‐term complete remission of multiple extranodal natural killer/T‐cell‐type posttransplant lymphoproliferative disorder after surgical resection: a case report. Transplant Proc. 2014;46:2373‐2376. 25011572 10.1016/j.transproceed.2014.02.014

[7] Seo E , Kim J , Oh SH , Kim KM , Kim DY , Lee J . Epstein‐Barr viral load monitoring for diagnosing post‐transplant lymphoproliferative disorder in pediatric liver transplant recipients. Pediatr Transplant. 2020;24:e13666. 32067332 10.1111/petr.13666

[8] Huang JG , Tan MYQ , Quak SH , Aw MM . Risk factors and clinical outcomes of pediatric liver transplant recipients with post‐transplant lymphoproliferative disease in a multi‐ethnic Asian cohort. Transplant Infect Dis. 2018;20. doi:10.1111/tid.12798 29071779

[9] Tajima T , Hata K , Haga H , et al. Post‐transplant lymphoproliferative disorders after liver transplantation: a retrospective cohort study including 1954 transplants. Liver Transplant. 2021;27:1165‐1180. 10.1002/lt.26034 PMC8453854 33655645

[10] Peters AC , Akinwumi MS , Cervera C , et al. The changing epidemiology of posttransplant lymphoproliferative disorder in adult solid organ transplant recipients over 30 years: a single‐center experience. Transplantation. 2018;102:1553‐1562. 29485513 10.1097/TP.0000000000002146

[11] Styczynski J , Reusser P , Einsele H , et al. Management of HSV, VZV and EBV infections in patients with hematological malignancies and after SCT: guidelines from the Second European Conference on Infections in Leukemia. Bone Marrow Transplant. 2009;43:757‐770. 19043458 10.1038/bmt.2008.386

[12] Styczynski J , van der Velden W , Fox CP , et al. Management of Epstein‐Barr Virus infections and post‐transplant lymphoproliferative disorders in patients after allogeneic hematopoietic stem cell transplantation: Sixth European Conference on Infections in Leukemia (ECIL‐6) guidelines. Haematologica. 2016;101:803‐811. 27365460 10.3324/haematol.2016.144428 PMC5004459

[13] Wareham NE , Mocroft A , Sengeløv H , et al. The value of EBV DNA in early detection of post‐transplant lymphoproliferative disorders among solid organ and hematopoietic stem cell transplant recipients. J Cancer Res Clin Oncol. 2018;144:1569‐1580. 29804164 10.1007/s00432-018-2674-9 PMC11813311

[14] Hourigan MJ , Doecke J , Mollee PN , et al. A new prognosticator for post‐transplant lymphoproliferative disorders after renal transplantation. Br J Haematol. 2008;141:904‐907. 18422782 10.1111/j.1365-2141.2008.07149.x

[15] Evens AM , David KA , Helenowski I , et al. Multicenter analysis of 80 solid organ transplantation recipients with post‐transplantation lymphoproliferative disease: outcomes and prognostic factors in the modern era. J Clin Oncol. 2010;28:1038‐1046. 20085936 10.1200/JCO.2009.25.4961 PMC2834429

[16] Montanari F , Radeski D , Seshan V , Alobeid B , Bhagat G , O'Connor OA . Recursive partitioning analysis of prognostic factors in post‐transplant lymphoproliferative disorders (PTLD): a 120 case single institution series. Br J Haematol. 2015;171:491‐500. 26250758 10.1111/bjh.13621

[17] Narkewicz MR , Green M , Dunn S , et al. Decreasing incidence of symptomatic Epstein‐Barr virus disease and posttransplant lymphoproliferative disorder in pediatric liver transplant recipients: report of the studies of pediatric liver transplantation experience. Liver Transplant. 2013;19:730‐740. 10.1002/lt.23659 PMC5001558 23696264

[18] Khedmat H , Taheri S . Early versus late outset of lymphoproliferative disorders post‐heart and lung transplantation: the PTLD. Int Survey Hematol Oncol Stem Cell Ther. 2011;4:10‐16. 21460602 10.5144/1658-3876.2011.10

[19] Lo RC , Chan SC , Chan KL , Chiang AK , Lo CM , Ng IO . Post‐transplant lymphoproliferative disorders in liver transplant recipients: a clinicopathological study. J Clin Pathol. 2013;66:392‐398. 23423516 10.1136/jclinpath-2012-201139

[20] Luskin MR , Heil DS , Tan KS , et al. The impact of EBV status on characteristics and outcomes of posttransplantation lymphoproliferative disorder. Am J Transplant. 2015;15:2665‐2673. 25988622 10.1111/ajt.13324 PMC5726526

[21] Cowles RA , Yahanda AM . Laparoscopic biopsy of abdominal retroperitoneal lymphadenopathy for the diagnosis of lymphoma. J Am Coll Surg. 2000;191:108‐113. 10898191 10.1016/s1072-7515(00)00279-9

[22] Diulus L , Chalikonda S , Pitt T , Rosenblatt S . Efficacy of laparoscopic mesenteric/retroperitoneal lymph node biopsy. Surg Endosc. 2009;23:389‐393. 18461391 10.1007/s00464-008-9935-7

[23] Green M , Soltys K , Rowe DT , Webber SA , Mazareigos G . Chronic high Epstein‐Barr viral load carriage in pediatric liver transplant recipients. Pediatr Transplant. 2009;13:319‐323. 18397216 10.1111/j.1399-3046.2008.00926.x

[24] Cho YU , Chi HS , Jang S , Park SH , Park CJ . Pattern analysis of Epstein‐Barr virus viremia and its significance in the evaluation of organ transplant patients suspected of having posttransplant lymphoproliferative disorders. Am J Clin Pathol. 2014;141:268‐274. 24436276 10.1309/AJCP9WYEXKOL9YUV

[25] Yancoski J , Danielian S , Ibañez J , et al. Quantification of Epstein‐Barr virus load in Argentinean transplant recipients using real‐time PCR. J Clin Virol. 2004;31:58‐65. 15288615 10.1016/j.jcv.2004.02.015

[26] Chen HS , Ho MC , Hu RH , et al. Roles of Epstein‐Barr virus viral load monitoring in the prediction of posttransplant lymphoproliferative disorder in pediatric liver transplantation. J Formosan Med Assoc. 2019;118:1362‐1368. 30612881 10.1016/j.jfma.2018.12.007

[27] Fox CP , Burns D , Parker AN , et al. EBV‐associated post‐transplant lymphoproliferative disorder following in vivo T‐cell‐depleted allogeneic transplantation: clinical features, viral load correlates and prognostic factors in the rituximab era. Bone Marrow Transplant. 2014;49:280‐286. 24212561 10.1038/bmt.2013.170

[28] van Esser JW , Niesters HG , van der Holt B , et al. Prevention of Epstein‐Barr virus‐lymphoproliferative disease by molecular monitoring and preemptive rituximab in high‐risk patients after allogeneic stem cell transplantation. Blood. 2002;99:4364‐4369. 12036863 10.1182/blood.v99.12.4364

[29] Patriarca F , Medeot M , Isola M , et al. Prognostic factors and outcome of Epstein‐Barr virus DNAemia in high‐risk recipients of allogeneic stem cell transplantation treated with preemptive rituximab. Transplant Infect Dis. 2013;15:259‐267. 10.1111/tid.12061 23405972

[30] Carpenter B , Haque T , Dimopoulou M , et al. Incidence and dynamics of Epstein‐Barr virus reactivation after alemtuzumab‐based conditioning for allogeneic hematopoietic stem‐cell transplantation. Transplantation. 2010;90:564‐570. 20555307 10.1097/TP.0b013e3181e7a3bf

[31] Kobayashi S , Sano H , Mochizuki K , et al. Pre‐emptive rituximab for Epstein‐Barr virus reactivation after haplo‐hematopoietic stem cell transplantation. Pediatrics Int. 2017;59:973‐978. 10.1111/ped.13336 28581032

